# An Alternative Treatment of Pseudogynecomastia in Male Patients After Massive Weight Loss

**DOI:** 10.1093/asjof/ojaa013

**Published:** 2020-05-16

**Authors:** Krista L Hardy, Ran Stark, Kevin H Small, Jeffrey M Kenkel

## Abstract

**Background:**

There has been an increase in body contouring procedures following massive weight loss (MWL), including male breast reduction procedures. Treating male chest deformity after MWL using standard mastopexy techniques often leads to suboptimal results.

**Objectives:**

The authors describe a technique to treat pseudogynecomastia using a modified elliptical excision and nipple-areola complex (NAC) transposition on a thinned inferior dermal pedicle as an alternative to conventional techniques.

**Methods:**

A retrospective chart review from January 2011 to January 2019 identified a total of 14 male patients who underwent excision of pseudogynecomastia using the described technique.

**Results:**

Patients were characterized by age, method of weight loss, pre-weight loss body mass index (BMI), post-weight loss BMI, total weight loss, grade of pseudogynecomastia, and concurrent procedures performed. Patients were followed for a period ranging from 3 months to 1.5 years (average, 8.1 months). Pre-weight loss BMI and post-weight loss BMI averaged 52.0 kg/m^2^ and 29.6 kg/m^2^, respectively. The average weight lost was 79.72 kg and the average total amount of tissue removed was 2615 g. All patients had concurrent procedures with an average operative time of 274 minutes. Four out of 14 patients (28.6%) experienced minor complications, which included asymmetry, delayed wound healing, seroma, and hyperpigmentation. There were no wound infections, hematomas, flap necrosis, or dysesthesia.

**Conclusions:**

Due to several cosmetic advantages and low complication profile, our technique using a modified elliptical excision and NAC transfer on an inferior dermal pedicle is an attractive option for treating male chest deformity after MWL.

**Level of Evidence: 4:**

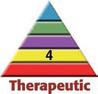

The demand for body contouring procedures following massive weight loss (MWL) has increased in response to the increase in bariatric surgery.^1^ Breast deformities after MWL are common in males, who comprise up to 20% of patients undergoing bariatric surgery.^2,3^ In 2018, the American Society for Aesthetic Plastic Surgery reported a total of 24,672 male breast reduction procedures, now ranked as the 13th most common cosmetic surgical procedure.^4^ Patients present with pseudogynecomastia, defined by retained retroareolar fat without glandular hypertrophy,^5^ often with excess tissue and skin anteriorly and laterally. Treating this deformity can be challenging, and the use of standard mastopexy techniques in male MWL patients commonly leads to suboptimal results.

Standard surgical treatment of excess male breast tissue began with the intra-areolar semicircular approach after its characterization by Webster in 1946.^6^ Although effective for smaller breasts, this technique was insufficient for larger deformities, especially those with significant ptosis and redundancy. To address these issues, later approaches employed a transverse elliptical excision with repositioning of the nipple-areola complex (NAC), which is accomplished either by transposing it on a pedicle^7,8^ or by performing a full-thickness graft.^9,10^ The addition of suction-assisted lipectomy^11^ and subsequently ultrasound-assisted liposuction as an adjunct to excision allowed for improvements in contouring.^12^ Various other methods, many adapted from reduction mammaplasty techniques designed for women, have been used with variable success, including circumareolar, inverted-T, and vertical approaches.^13^ More recently, treatment of chest deformity after MWL has been extended to include the lateral chest wall using a widened elliptical excision with either a pedicled or free NAC transfer.^14-17^

There continues to be drawbacks to the current treatment of pseudogynecomastia. The pedicled technique has been criticized for its bulkiness, resulting in feminization of the male chest.^14^ This can be avoided with free NAC grafting; however, this approach comes with the disadvantages of dyspigmentation and a deflated or stuck-on appearance.^18^ To address these challenges, the authors describe a technique consisting of a modified elliptical excision and NAC transposition on an inferior dermal pedicle to avoid excessive bulk.

## METHODS

This retrospective review was approved by the Institutional Review Board at UT Southwestern. From January 2011 to January 2019, a total of 14 consecutive male weight loss patients (patients A-N) who underwent excision of pseudogynecomastia using the below described technique were identified. 

**Figure 1.**
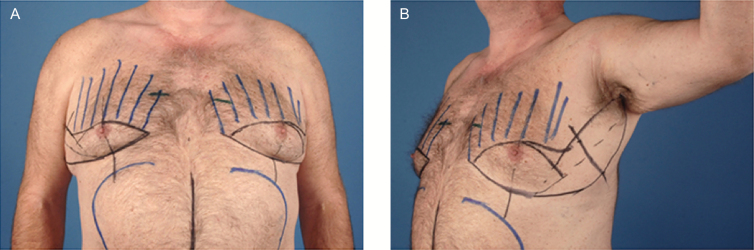
Surgical markings. A 57-year old male seen preoperatively for excision of pseudogynecomastia in (A) frontal view and (B) oblique view. Markings indicate planned elliptical incision with extension toward the axilla just lateral to the anterior axillary fold to remove lateral chest excess.

The male breast lift technique is ideal for a patient with a NAC that is on the lower pole or below the normal inframammary fold (IMF). Transposition of the IMF onto the chest confirms that excision at or above the NAC can occur (lift and drop technique; Video 1). This excisional pattern is designed as an ellipse incorporating the NAC. The width of dermal pedicle is based on the entire IMF incision with extension approximately 1 cm above the NAC. In cases with a lateral chest deformity, the excision is extended up toward the axilla along the anterior axillary fold and often down the arm when brachioplasty is simultaneously performed. The anterior incision laterally is planned lateral to the anterior axillary fold helping to conceal it. The posterior incision is marked with a lift and drop in a superior and medial vector and will be confirmed during the procedure (Figure 1).

A wetting solution consisting of 1 L of lactated ringers and 1 ampule of 1:1000 epinephrine is infiltrated for hemostatic control. Based on the initial markings, an elliptical incision is made at the superior aspect and at the new IMF. The dermal pedicle is deepithelialized preserving the new NAC. The NAC is measured to be approximately 20 to 25 mm in diameter.

**Figure 2.**
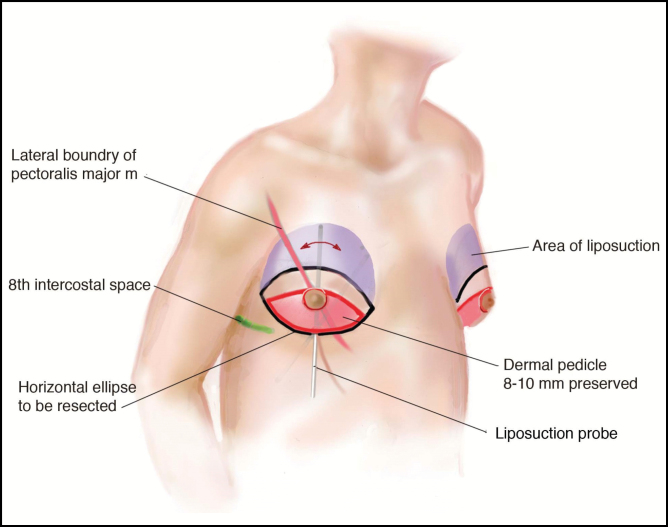
Illustration of preoperative markings and pertinent reference points.

Liposuction is then performed. The goal of liposuction in the male breast lift is to create a smooth transition from the superior chest to the inferior chest as well as soften the IMF crease. Access sites can be placed at the discretion of the surgeon but should be small not to disrupt the vascular supply. Our preference is along the inferior border of the dermal pedicle, medially and laterally (Figure 2).

**Figure 3.**
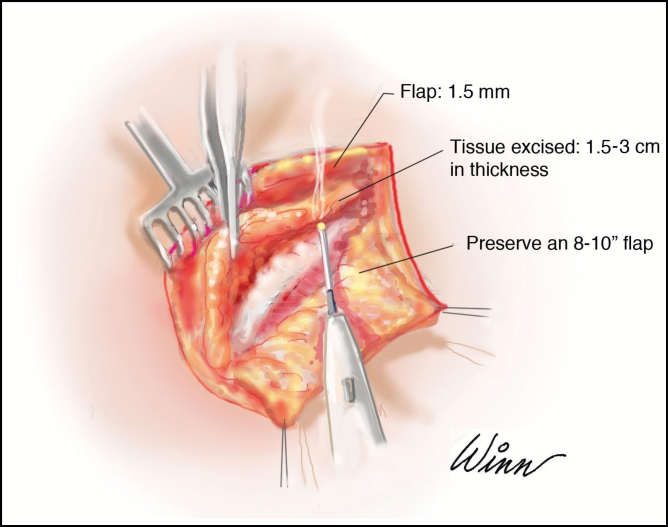
Intraoperative illustration showing the dermal pedicle reflected caudally depicting the plane of dissection above and below the gland.

A dermal pedicle is elevated maintaining approximately 5 to 10 mm of fat subdermally for vascular preservation. Preserving a wide dermal pedicle keeps the base of the random flap wide helping to maintain an appropriate blood supply, with main contributions from the internal mammary and lateral thoracic arteries. Once elevated, the remaining tissue is first dissected superiorly transitioning at a 45-degree angle toward the underlying *pectoralis* major. This area has been treated previously with liposuction allowing for ease of dissection and a smooth transition point. The excess breast tissue remaining over the *pectoralis* major fascia down to the IMF is excised (Figure 3).

Once hemostasis is confirmed, the pedicle is secured to the chest wall at its base, medially and laterally, using an absorbable braided suture. This fixation prevents the edges from overturning, reduces tension, and allows mobility superior for NAC inset. The superior skin flap is draped over the dermal pedicle and temporarily secured.

When needed, the resection of the lateral chest can begin (when appropriate). The anterior incision is made and the dissection is carried down to and through Scarpa’s fascia. The dissection is carried posteriorly toward the posterior mark. Care is taken to stay lateral to the border of the *latissimus dorsi* muscle in a more superficial plane. The lateral chest wall fasciocutaneous flap is undermined, transposed in the superior medial vector, and confirmed for resection. Three-point sutures using an absorbable, longer-lasting monofilament suture, are used to secure the lateral and medial skin flaps to the chest wall at the anterior incision point. The 3-point suture is used to prevent migration of the incision line, preserving it posterior to the anterior axillary line and avoiding lateralization of the breast complex and should provide rigid fixation.

Whether a lateral chest excision is performed or not, the lateral IMF must be lifted and repositioned as it often descends disproportionately from the medial attachments. The placement and final location of the IMF are secondary to the debulking and removal of the majority of the anterior fullness.

The final position of the new NAC is determined by the intersection of the lateral border of the *pectoralis* major and fourth intercostal space and is confirmed in the upright position during surgery. While it is always the desired outcome to have the IMF on each side precisely symmetric, this can be difficult particularly when dealing with lax, descended tissue. In general, we accept up to a 1 cm variance between the 2 sides. If a slight asymmetry exists, it may make confirmation of NAC symmetry more difficult and should be accounted for. An 18- mm circular pattern is marked and deepithelialized. Preservation of the dermal shelf for the NAC helps stabilize and acts as a backup if there is a vascular compromise of the NAC and a free nipple graft is needed. The NAC is delivered through the incision and is secured with monofilament absorbable sutures.

We use liposomal Bupivacaine (Exparel, San Diego, CA) for prolonged pain control. The dermis is closed in layers using an absorbable monofilament. Barbed suture is used for closure, and a meshed tape with a topical glue (Prineo, Ethicon, NJ) is used to dress the incision line. A vest garment provides compression of the chest. There is no movement restriction; however, heavy physical activity is limited for the first 3 weeks.

## RESULTS

Patients were characterized by age, method of weight loss, pre-weight loss body mass index (BMI), post-weight loss BMI, total weight loss, grade of pseudogynecomastia, and concurrent procedures performed (Table 1). Patients were between the ages of 31 and 58 years with an average age of 40.5 years. The average for pre-weight loss BMI and post-weight loss BMI were 52.0 kg/m^2^ (range, 34.5-91.2 kg/m^2^) and 29.6 kg/m^2^ (range, 25.1-36.2 kg/m^2^), respectively. Average weight loss was 79.72 kg (range, 25.0-186.0 kg). Three patients were former smokers but quit several years before surgery. None of the patients had undergone breast surgery previously.

**Table 1.** Patient Characteristics

Patient	Age, years	Pre-WL BMI, kg/m^2^	Post-WL BMI, kg/m^2^	Total-WL, kg	Method-WL	Grade^a^	Other procedures
A	31	66.1	31.8	111.58	Gastric sleeve	II	Brachioplasty, thighplasty
B	39	49.3	30.1	58.97	Lifestyle modification	II	Lower body lift, thighplasty
C	57	44.2	32.7	36.28	Gastric sleeve	IB	Abdominoplasty
D	45	91.2	29.0	185.98	RYGB	III	Abdominoplasty, brachioplasty, neck z-plasty
E	34	53.3	30.8	79.40	Band	III	Abdominoplasty
F	34	39.9	25.1	50.36	Lifestyle modification	II	Abdominoplasty
G	37	47.4	29.5	70.30	Lifestyle modification	IB	Lower body lift, thighplasty
H	38	61.0	25.5	125.20	Lifestyle modification	II	Abdominoplasty, brachioplasty
I	58	41.1	30.2	38.50	Lifestyle modification	—	Abdominoplasty
J	30	58.1	31.2	90.00	Gastric sleeve	—	Panniculectomy
K	44	54.3	28.6	81.65	Gastric sleeve	III	Upper body lift, abdominoplasty, brachioplasty
L	32	34.5	27.5	25.00	Lifestyle modification	—	Lower body lift
M	52	42.4	26.7	131.2	Gastric sleeve	I	Abdominoplasty
N	36	44.7	36.2	31.8	Lifestyle modification	—	Abdominoplasty

RYGB, Roux Y Gastric Bypass; WL, weight loss.

^a^Grade of pseudogynecomastia based on classification by Gusenoff et al.^14^

**Figure 4.**
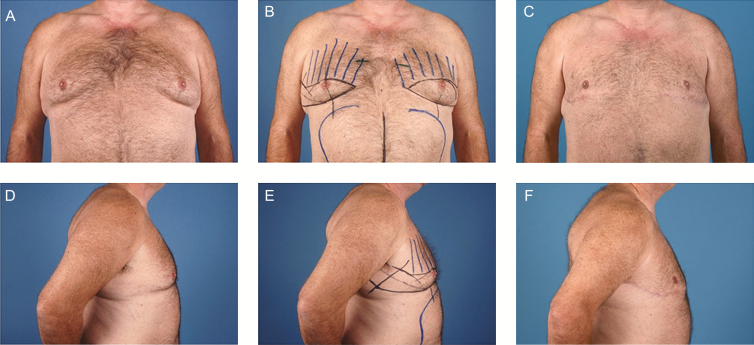
Patient C, a 57-year-old male with grade 1B pseudogynemastia after gastric sleeve resulting in a 36 kg sustained weight loss. (A, B, D, E) Preoperative and (C, F) 12 months following anterior and lateral chest excision with abdominoplasty.

**Figure 5.**
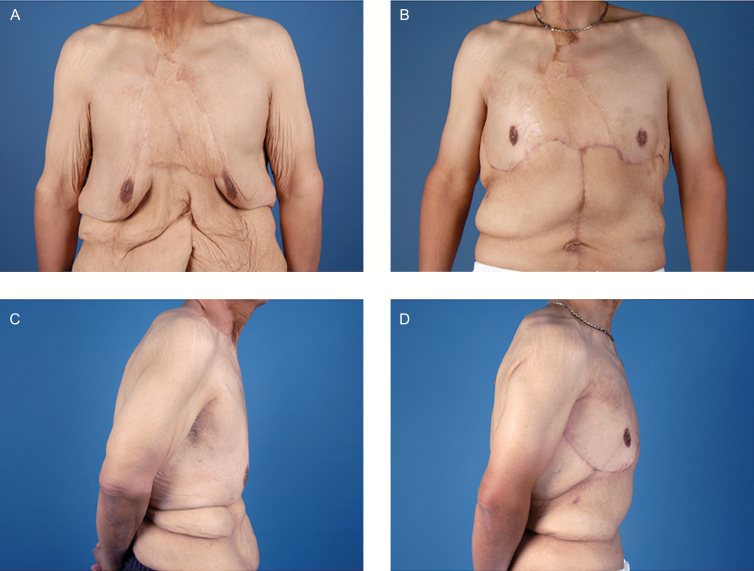
Patient with severe pseudogynecomastia. Patient D, a 45-year-old male with grade III pseudogynecomastia after a laparoscopic Roux-en-Y gastric bypass resulting in a 185.98-kg weight loss. (A, C) Preoperative and (B, D) postoperative views are shown. The postoperative photographs were taken 5 months after surgery. Note history of burn scars and skin grafting on the patient.

Preoperative photographs were used to classify the severity of pseudogynecomastia based on the system devised by Gusenoff et al.^14^ The authors define grade 1 pseudogynecomastia by minimal displacement of the NAC and IMF, further distinguished by the absence (1a) or presence (1b) of a lateral chest roll. Grade 2 includes those with significant descent of the NAC and IMF, presence of a lateral roll, and minimal upper abdominal laxity. Grade 3 deformities are similar to grade 2, except they are characterized by significant upper abdominal laxity.^14^ Postoperative photographs included follow-up between 3 months to 1.5 years postoperatively, with an average follow-up of 8.1 months (Figures 4 and 5).

All patients who underwent excision of pseudogynecomastia also underwent simultaneous procedures, with abdominoplasty being the most common. The average operative time was 274 minutes and the average total amount of tissue removed was 2615 g. Length of stay was usually 1 day.

**Figure 6.**
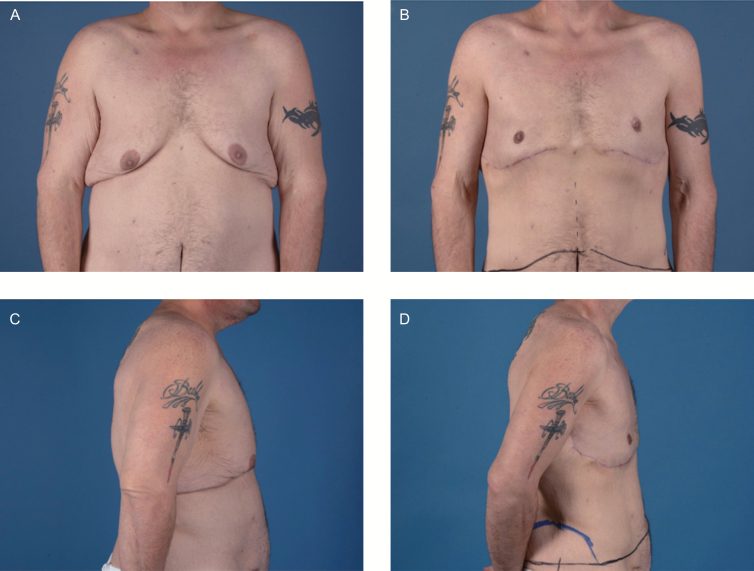
Patient with postoperative nipple asymmetry. Patient A, a 31-year-old male with grade II pseudogynecomastia. (A, C) Preoperative and (B, D) postoperative views are shown. The postoperative photographs were taken 3 months after surgery. He developed asymmetry of the nipple-areola complexes, with the left nipple placed higher than the right.

**Figure 7.**
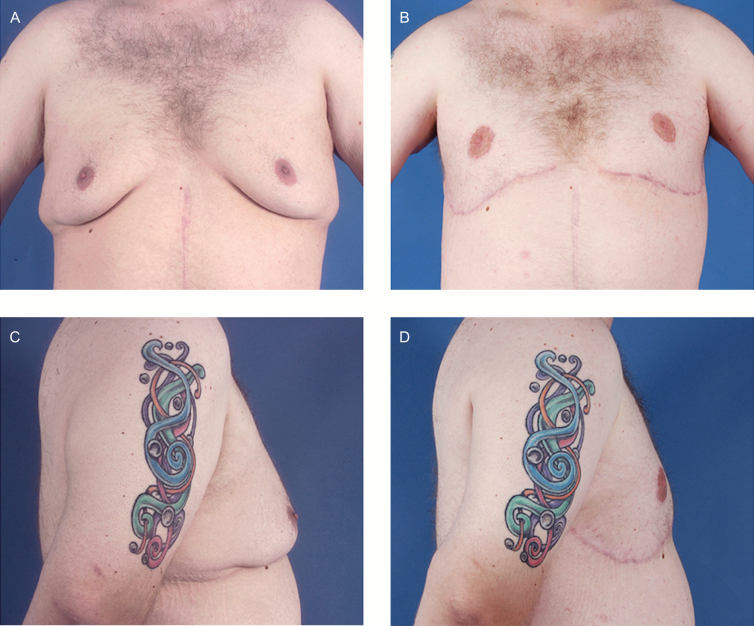
Patient with conversion to free nipple graft early in our experience. Patient H, a 38-year-old male with grade II pseudogynecomastia. (A, C) Preoperative and (B, D) postoperative views are shown. The postoperative photographs were taken 6 months after surgery demonstrates the flat and large appearance of the nipple after conversion to free nipple graft.

Four out of 14 patients (28.6%) experienced complications (Table 2). Asymmetry of the NACs was seen in patient A, who was satisfied with the result and did not pursue revision (Figure 6). Patient D had delayed wound healing and left central nipple necrosis that resolved without requiring further intervention. In patient E, who was African American, there was mild hyperpigmentation of the NAC and asymmetry of the inframammary folds with the left placed higher than the right. No revision was performed. Patient L developed a seroma to the right lateral back which resolved after drainage in the office. There were no wound infections, hematomas, flap necrosis, or dysesthesia in our patient population. Of note, patient H had poor nipple perfusion intraoperatively and was converted to free nipple graft (Figure 7).

**Table 2.** Complications

Patient	Grade	Complications	Revision
A	II	NAC asymmetry	None
B	II	None	None
C	IB	None	None
D	III	Delayed healing, nipple necrosis	None
E	III	Hyperpigmentation, IMF asymmetry	None
F	II	None	None
G	IB	None	None
H	IB	None	None
I	—	None	None
J	—	None	None
K	III	None	None
L	—	Seroma	None
M	I	None	None
N	—	None	None

## DISCUSSION

Pseudogynecomastia seen after MWL is characterized by increased retroareolar fat without mammary hypertrophy, in contrast to gynecomastia, which is most often idiopathic and results in proliferation of glandular tissue.^19^ Treatment for pseudogynecomastia generally requires excision of excess tissue and skin for all but the mildest forms, which can have excellent results with liposuction alone due to minimal skin redundancy and displacement of the NAC. A multitude of excisional procedures for the treatment of gynecomastia have been applied to pseudogynecomastia.^20-22^ Excisional techniques first developed for female breast lift have been used in males with limited success, often resulting in unfavorable scars, asymmetry, and a feminized chest.^13^ Concentric circle or circumareolar approaches with or without purse-string closure may result in corrugated scars or residual skin excess.^23^ The most widely used method employs a horizontal ellipse pattern,^14-17^ which is better for recreating the ideal contour of the male chest and produces a scar that closely approximates the IMF.^14,15,17^ The NAC has been either transposed on a dermoglandular pedicle or transferred as a full-thickness graft. Horizontally based excisional patterns with free nipple graft remain the gold standard in most cases of pseudogynecomastia. 

Pseudogynecomastia has predominantly been classified with the most severe categories of gynecomastia^24-26^; however, there are a variety of defects present in MWL patients that traditional classifications and treatment algorithms do not address. Gusenoff et al^14^ have proposed a classification scheme and a treatment algorithm for pseudogynecomastia that accounts for these unique features. Based on the algorithm, grade 1 pseudogynecomastia is treated with liposuction of the anterior chest and direct excision of the lateral roll, if present. Patients with higher grades undergo horizontal elliptical excision and extension to the axilla to include the lateral roll, with grade 2 deformities receiving pedicled reconstruction and grade 3 receiving free nipple grafts. The authors believe that the significant upper abdominal laxity found in grade 3 pseudogynecomastia may result in an unreliable pedicle, which is why they recommend grafting in these patients. Pedicled reconstruction is more time consuming and presents a greater risk of flap and nipple necrosis.^18^ However, experts in their study related the pedicled technique to better NAC aesthetics, as free grafts often have an unnatural, flat appearance, increased scarring, and pigmentation changes.

A widely based inferior pedicle, however, may lead to poor outcomes in patients with long nipple-to-IMF distances, creating a bulky chest, causing venous congestion, or resulting in flap necrosis due to the increased length of the pedicle. In these cases, the NAC can be transferred on a superior pedicle, although this places the scar above the nipple rather than at the inferior border of the *pectoralis* major.^16^ Alternatively, a technique introduced by Stoff et al^15^ employs a central mound, which can be used in patients with severely ptotic breasts while also allowing for ideal scar placement. Other variations include a superolateral pedicle designed to give lateral fullness, providing a broader chest and mimicking the lateral border of the *pectoralis* major.^17^ These described techniques leave the patients with undesirable tissue bulk, often feminizing or failing to address the concerns of the patient. In these cases, free nipple graft may be a reasonable alternative despite the dyspigmentation in some cases.

The method, we propose, provides many benefits to conventional mastopexy. The dermal pedicle described allows for preservation of the vascularity of the NAC and minimal bulk to the anterior chest. This is particularly relevant in darker skin types where free nipple grafts may result in dyspigmentation. Extension laterally allows for simultaneous correction of lateral chest deformity often seen after significant weight loss. The positioning of the scar in the IMF is preferred over a vertical scar because it follows the natural contours of the male chest, corresponding to the inferior border of the *pectoralis* major. The lateral scar, placed posteriorly to the anterior axillary line, is hidden beneath the adducted arm. One limitation of the transverse ellipse pattern is that it removes mostly vertical redundancy. Hurwitz^27^ addresses this issue with a boomerang incision created by asymmetrical elliptical incisions placed at right angles to one another just above the NAC. This may be combined with a J torsoplasty to correct both vertical and horizontal laxity in the chest and upper abdomen. However, this method creates an interrupted sinusoidal scar pattern that is not as aesthetically pleasing as the horizontal ellipse. Thus, it should only be used in patients who can accept an undulating scar in return for minimal residual skin laxity. We have found that horizontal excision unnecessary using this technique.

The technique described is based on a random, inferior dermal pedicle. Excessive projection is avoided by carefully thinning the pedicle and aggressively resecting the remaining tissue on the chest. Liposuction can be a useful adjunct.

When combing chest excision with abdominoplasty, we tend to perform the chest excision first allowing more positioning flexibility. To prevent scar migration and descent, we stabilize the IMF medially and laterally with deep sutures. It is our belief that that the majority of weight loss patients have a prominent costal margin with excessive distances from the IMF to costal margin making descent less of an issue.

One patient in our study did have partial nipple necrosis; however, this resolved without further intervention. The proposed technique otherwise has an acceptable complication profile, with only 1 patient developing seroma. Although Gusenoff et al^14^ found that 3 out of 8 patients with pedicled reconstructions experienced dysesthesia, there were no reported dysesthesia in our group, suggesting that the neurovascular supply of the NAC was relatively well preserved, even in the most severe grades of pseudogynecomastia.

Although our study remains the second largest investigation to date describing the use of a dermal pedicle for male breast lift after MWL,^14-17^ we are limited by a small sample size and single-surgeon experience. As a subjective review, assessment of the outcomes may be biased, and future studies should focus on patient satisfaction and objective measures of cosmetic appearance. Moreover, we do not directly compare our technique to others, including the use of free nipple graft.

## CONCLUSIONS

Pseudogynecomastia can be managed with a novel technique utilizing a modified elliptical excision with J extension and NAC transfer on a thin, inferior dermal pedicle. This method may be more acceptable in ethnic patients concerned about dyspigmentation issues that may follow standard free nipple graft techniques. This technique offers a low complication profile, making it an attractive option for the growing population of male patients seeking body contouring after MWL.
